# CCAAT/Enhancer Binding Protein β inhibits myogenic differentiation via ID3

**DOI:** 10.1038/s41598-018-34871-0

**Published:** 2018-11-09

**Authors:** Hamood AlSudais, Neena Lala-Tabbert, Nadine Wiper-Bergeron

**Affiliations:** 1 Graduate Program in Cellular and Molecular Medicine, Faculty of Medicine, University of Ottawa, 451 Smyth Road, Ottawa, Ontario K1H 8M5 Canada; 2 Department of Cellular and Molecular Medicine, Faculty of Medicine, University of Ottawa, 451 Smyth Road, Ottawa, Ontario K1H 8M5 Canada

## Abstract

Myogenesis is regulated by the coordinated expression of muscle regulatory factors, a family of transcription factors that includes MYOD, MYF5, myogenin and MRF4. Muscle regulatory factors are basic helix-loop-helix transcription factors that heterodimerize with E proteins to bind the regulatory regions of target genes. Their activity can be inhibited by members of the Inhibitor of DNA binding and differentiation (ID) family, which bind E-proteins with high affinity, thereby preventing muscle regulatory factor-dependent transcriptional responses. CCAAT/Enhancer Binding protein beta (C/EBPβ) is a transcription factor expressed in myogenic precursor cells that acts to inhibit myogenic differentiation, though the mechanism remains poorly understood. We identify *Id3* as a novel C/EBPβ target gene that inhibits myogenic differentiation. Overexpression of C/EBPβ stimulates *Id3* mRNA and protein expression, and is required for C/EBPβ-mediated inhibition of myogenic differentiation. Misexpression of C/EBPβ in myogenic precursors, such as in models of cancer cachexia, prevents the differentiation of myogenic precursors and we show that loss of *Id3* rescues differentiation under these conditions, suggesting that the stimulation of *Id3* expression by C/EBPβ is an important mechanism by which C/EBPβ inhibits myogenic differentiation.

## Introduction

Skeletal muscle tissue retains the ability to regenerate throughout the lifespan of an organism and this ability is dependent on the presence of a small population of skeletal muscle stem cells called satellite cells. Normally quiescent, in response to injury satellite cells can become activated to proliferate, differentiate and fuse to repair damaged muscle^1,2^. Myogenic differentiation is controlled by a group of four basic helix-loop-helix (bHLH) transcription factors called muscle regulatory factors (MRFs) whose coordinated expression drives muscle-specific gene expression^3,4^. MYF5 and MYOD are expressed during activation and proliferation of satellite cells and their expression is followed by myogenin (MYOG) and MRF4 which drive terminal differentiation^5^. To exert their actions, MRFs heterodimerize with E-proteins to bind the regulatory regions of their target genes.

The activity of the myogenic regulatory factors can be inhibited by members of the inhibitor of DNA binding and differentiation (ID) family^6–11^. The ID proteins, encoded by four genes (*Id1-4*), are members of the HLH superfamily; however, unlike bHLH transcription factors, they lack a DNA-binding domain and thus form inactive heterodimers with other bHLH members^6,7^. ID proteins bind strongly to E-proteins preventing their dimerization with MRFs and thereby inhibiting the activation of MRF target genes during myogenesis^6–9^. ID1, ID2 and ID3 can inhibit myogenic differentiation while ID4 is without effect^9,12–14^.

CCAAT/Enhancer Binding proteins (C/EBPs) are a family of six bzip transcription factors that are involved in the regulation of cellular functions such as apoptosis, differentiation and cell growth^15–17^. C/EBPβ plays a significant role in the regulation of mesenchymal stem cell fate including repression of osteoblastogenesis and stimulation of adipogenesis^17–20^. In addition to these roles, C/EBPβ is expressed in quiescent and proliferating skeletal muscle satellite cells and is rapidly decreased upon activation by the ubiquitin-proteasome system to allow for myogenic differentiation^21,22^. Overexpression of C/EBPβ in myoblasts blocks their differentiation, while loss of C/EBPβ expression promotes precocious differentiation and larger myotubes *in vitro*^22^. *In vivo*, loss of C/EBPβ in satellite cells results in larger myofibers and enhanced regenerative capacity after a single injury^22^. However, in this model, muscle regeneration was defective after a second injury due to impaired self-renewal and a reduction in the satellite cell pool supporting repair^23^. Although the role of C/EBPβ in satellite cell function has been elucidated, the specific mechanism by which C/EBPβ inhibits myogenic differentiation remains unknown.

C/EBPβ expression can interfere with the transcriptional activity of MYOD and has been shown to regulate the expression of *Id1* and *Id2* in the immune system^24–26^. In this study, we examined a potential role for ID proteins in the C/EBPβ-mediated inhibition of myogenic differentiation. We find that C/EBPβ stimulates *Id3* and *Id1* expression in differentiating myoblasts, but appears to only regulate *Id3* transcription directly. Knockdown of *Id3* or *Id1* rescues myogenic differentiation in cells overexpressing C/EBPβ, though neither could rescue fusion. Further, *Id3* is the most highly expressed Id family member in myoblasts, and only knockdown of ID3 could enhance myogenic differentiation in control cells.

## Results

### ID proteins are expressed in proliferating myoblasts and are downregulated with differentiation

**Figure 1:**
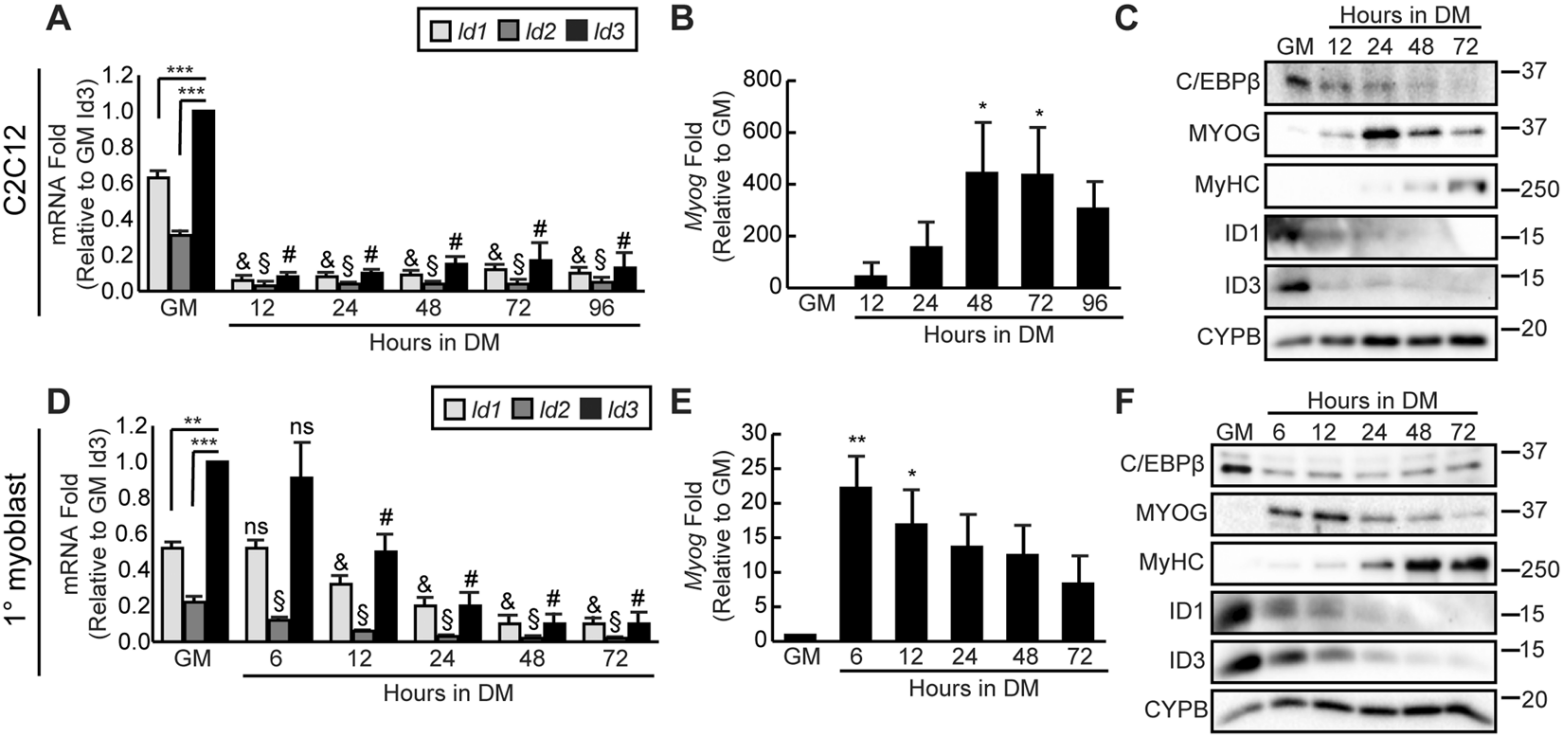
ID protein expression is regulated during myogenic differentiation. (**A**) RT-qPCR analysis of *Id1*, *Id2* and *Id3* expression in C2C12 myoblasts in growth medium (GM) and after induction to differentiate (DM). Data is presented as the fold change relative to *Id3* expression in GM. Bars are the mean ± SEM, n = 4, ***p < 0.001 for comparisons in GM. ^&^p < 0.0001 relative to *Id1* expression in GM. ^§^p < 0.0001 relative to *Id2* expression in GM. ^#^p < 0.0001 relative to *Id3* expression in GM. (**B**) *Myog* mRNA expression in cells cultured as in (A) presented as the fold change relative to GM condition. (**C**) C/EBPβ, myogenin (MYOG), myosin heavy chain (MyHC), ID1 and ID3 protein expression in C2C12 myoblasts cultured as in (A). Cyclophilin B (CYPB) is a loading control. (**D**) RT-qPCR analysis of *Id1*, *Id2* and *Id3* expression in proliferating (GM) and differentiating primary myoblasts (6–72 h). Data is presented as the fold change relative to *Id3* expression in GM. n = 3, **p < 0.01, ***p < 0.001 for comparisons in GM. ^&^p < 0.001 relative to *Id1* expression in GM. ^§^p < 0.001 relative to *Id2* expression in GM. ^#^p < 0.001 relative to *Id3* expression in GM. ns is not statistically significant relative to GM values. (**E**) *Myog* mRNA expression in primary myoblasts cultured as in (D) and presented as the fold change relative to its expression in GM. (**F**) C/EBPβ, myogenin (MYOG), myosin heavy chain (MyHC), ID1 and ID3 protein expression in primary myoblasts cultured as in (D).

ID1, ID2 and ID3 are known inhibitors of myogenic differentiation^9,12–14^. Their expression patterns in proliferating myoblasts and during myogenic differentiation were evaluated in the myoblast cell line C2C12 and in primary myoblasts isolated from mouse hindlimb muscle. In C2C12 cells, *Id1*, *Id2*, and *Id3* mRNAs were all detected in proliferating cells cultured in growth medium (Fig. 1A). All were rapidly and substantially downregulated 12 hours after induction to differentiate (Fig. 1A) and remained low up to 96 hours in differentiation medium. By contrast, myogenin (*Myog*) expression, a marker of terminal myogenic differentiation that is not expressed in proliferating myoblasts, was significantly induced after 48 hours in differentiation medium confirming differentiation (Fig. 1B). Assessment of protein expression patterns revealed that in accordance with their anti-myogenic roles, ID1, ID3 and C/EBPβ protein expression was highest in growth medium. Induction to differentiate caused a rapid downregulation of ID1 and ID3, evident 12 hours after switching to differentiation medium (Fig. 1C). C/EBPβ expression was also downregulated by 12 hours in differentiation medium, in accordance with published observations^21,22^. By contrast, myogenin expression was detected as early as 12 hours following induction to differentiate and levels peaked at the 24 hour time point (Fig. 1C). The structural protein, myosin heavy chain, was detected beginning at 24 hours after induction and continued to increase over the course of the experiment. ID2 protein levels were not examined as *Id2* mRNA was weakly expressed in comparison to *Id1* and *Id3* and a reliable antibody was not found (Fig. 1A).

While C2C12 myoblasts are a useful cell line to study myogenic differentiation, they are polyploid and have inactivation of the p19/Arf locus^27^ and thus do not always recapitulate events *in vivo*. Therefore we assessed ID expression in primary myoblasts. Consistent with our findings in C2C12 myoblasts, *Id1*, *Id2*, and *Id3* were all downregulated as early as 12 hours after induction to differentiate, with *Id2* expressed at lower levels (Fig. 1D). The time course of myogenic differentiation is condensed in primary myoblasts as compared to C2C12 cells with the expression of myogenin mRNA and protein levels upregulated 6 hours after the addition of differentiation media (Fig. 1E,F). ID1, ID3 and C/EBPβ protein, while high in growth medium, were rapidly downregulated 6 hours after induction of differentiation (Fig. 1F). Consistent with differentiation, myosin heavy chain expression increased after myogenin expression and following downregulation of ID1 and ID3 expression (Fig. 1F).

### C/EBPβ regulates the expression of Id3

**Figure 2:**
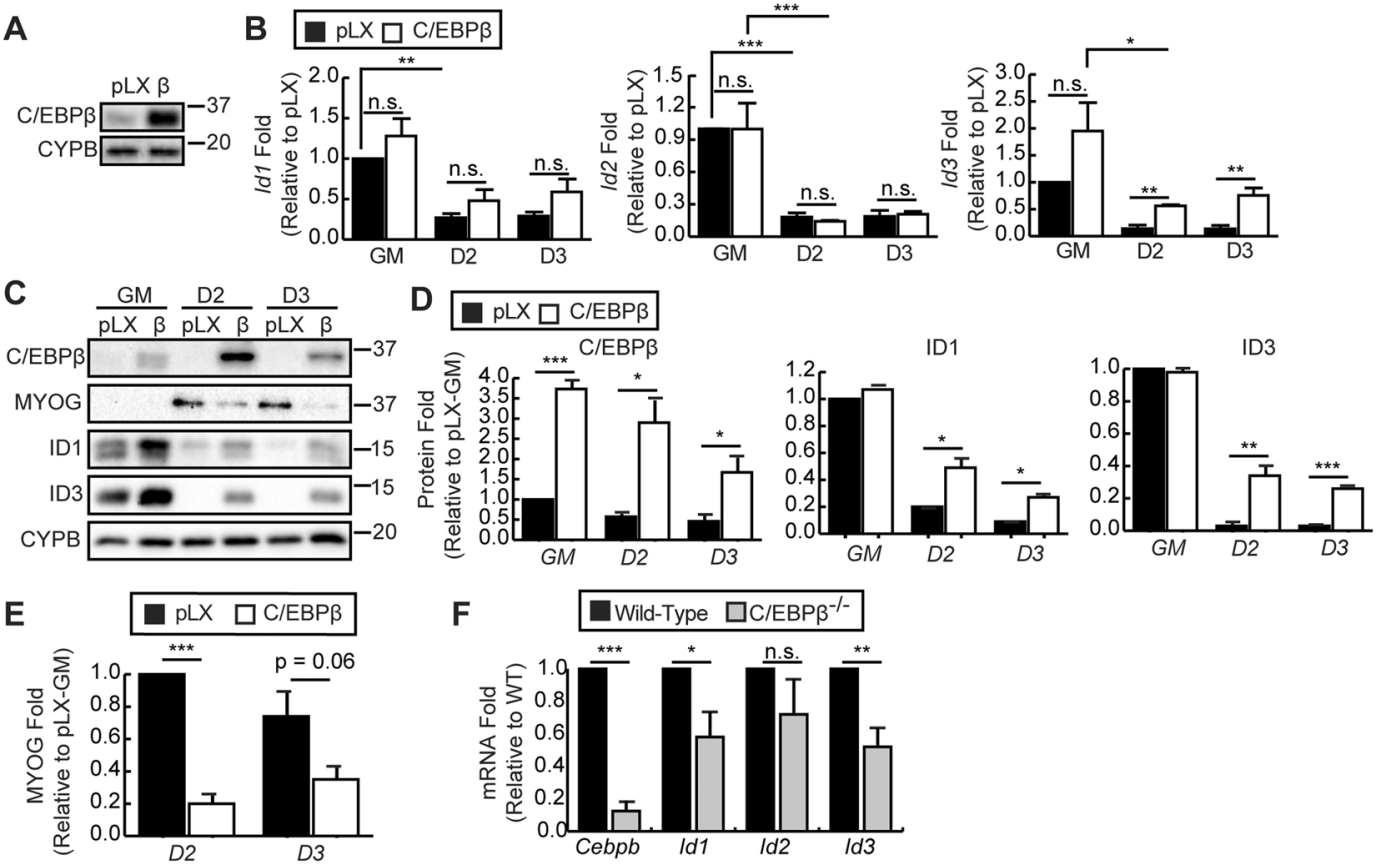
C/EBPβ regulates ID3 and ID1 expression. (**A**) C/EBPβ expression in proliferating C2C12 myoblasts that were retrovirally transduced with empty virus (pLX) or to express C/EBPβ (β). Cyclophilin B (CYPB) is a loading control. (**B**) *Id1*, *Id2* and *Id3* mRNA expression in cells transduced as in (A) in growth medium (GM) or cultured in differentiation medium for two (D2) or three days (D3) (n = 4). (**C**) C/EBPβ, myogenin (MYOG), ID1 and ID3 protein expression in C2C12 myoblasts transduced and cultured as in (B). (**D**) Quantification of C/EBPβ, ID1 and ID3 expression from (**D**) presented as fold change relative to pLX GM conditions after normalization to CYPB expression (n = 4). (**E**) Quantification of MYOG expression from (C) presented as fold change relative to pLXSN after two days (D2) of differentiation (n = 4). (**F**) *Cebpb*, *Id1*, *Id2* and *Id3* expression in proliferating primary myoblasts isolated from *Cebpb*^fl/fl^*Pax7*^wt/wt^ (wild-type) and *Cebpb*^fl/fl^*Pax7*^CreER/wt^ (C/EBPβ^−/−^) mice (n = 5). Bars are the mean ± SEM, *p < 0.05, **p < 0.01, ***p < 0.001, n.s. is not significant (two-tailed Student’s t-test or two-way ANOVA as required).

Given that C/EBPβ can inhibit myogenic differentiation and can regulate the expression of *Id1* and *Id2* in the immune system^24–26^, we examined *Id* gene expression in C2C12 myoblasts retrovirally transduced to express C/EBPβ (β) with an empty virus (pLXSN) under growth and differentiation conditions (Fig. 2). Overexpression of C/EBPβ was confirmed by western blot in proliferating cells (Fig. 2A). The expression of *Id1*, *Id2* and *Id3* in these cells was examined under growth and differentiation conditions (Fig. 2B). In proliferating cells, none of the ID genes were significantly upregulated. However, in differentiating cells, only *Id3* mRNA expression was increased in cells overexpressing C/EBPβ as compared to empty virus controls (Fig. 2B). However, western blot analysis revealed upregulation of ID1 and ID3 protein in differentiating C/EBPβ-overexpressing cells (Fig. 2C,D). Increased ID protein expression in C/EBPβ-overexpressing myoblasts correlated with a reduction in myogenin protein expression as compared to controls (Fig. 2C,E).

Conversely, in primary myoblasts isolated from a conditional null mouse where *Cebpb* is excised in muscle precursor cells (*Cebpb*^fl/fl^*Pax7*^CreER/wt^ (C/EBPβ^−/−^)), *Id1* and *Id3* mRNA expression were reduced as compared to non-Cre expressing littermate controls (*Cebpb*^fl/fl^*Pax7*^wt/wt^ (wild-type)) (Fig. 2F). The expression of *Id2* was not significantly affected by knockdown of *Cebpb* expression. Taken together, these data suggest that C/EBPβ regulates *Id1* and *Id3* expression in myoblasts.

### Loss of Id3 rescues C/EBPβ-mediated inhibition of myogenic differentiation

**Figure 3:**
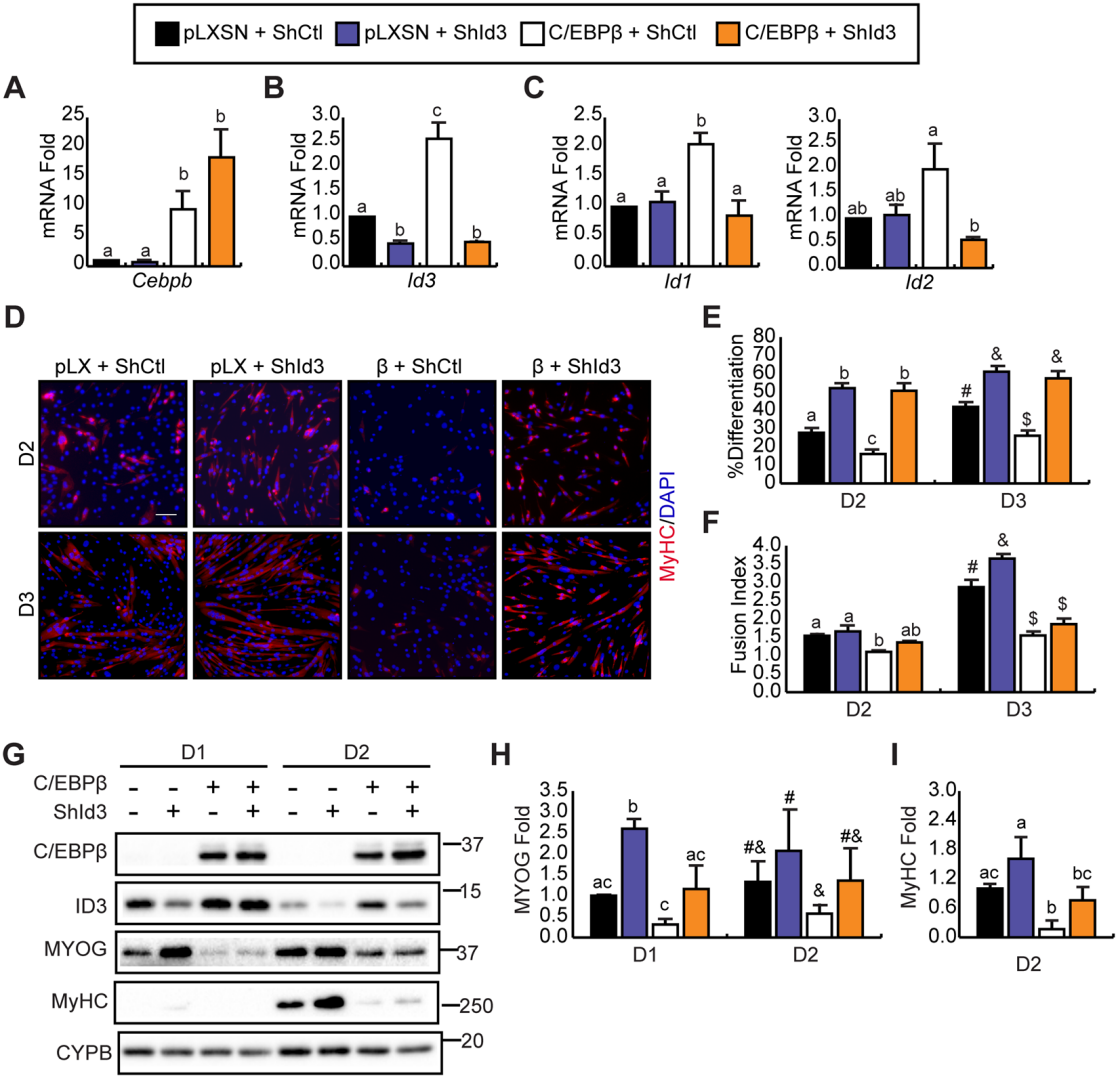
Inhibition of *Id3* expression rescues C/EBPβ-mediated inhibition of myogenic differentiation. C2C12 myoblasts expressing C/EBPβ or control plasmid (pLXSN) were retrovirally transduced to express a control shRNA (ShCtl) or a shRNA directed against *Id3* (ShId3) to create pooled stable cell lines. RT-qPCR analysis of (**A**) *Cebpb*, (**B**) *Id3*, and (**C**) *Id1* and *Id2* expression in myoblasts cultured in differentiation medium for two days. Data presented relative to pLXSN + ShCtl (n = 3). (**D**) MyHC (red) immunostaining of differentiating cells from (A) two (D2) and three (D3) days after induction. Nuclei are counterstained with DAPI (blue). Scale bar = 50 μm. (**E**) Differentiation index (# of MyHC^+^ cells relative to the total nuclei) from (**D**) (n = 3). (**F**) Fusion index (average # of nuclei per MyHC^+^ cell) (n = 3). (**G**) C/EBPβ, ID3, Myogenin (MYOG), myosin heavy chain (MyHC) protein expression in pooled stable myoblasts differentiated for one (D1) or two days (D2). Cyclophilin B (CYPB) is used as a loading control. (**H**) Quantification of MYOG protein expression from (G), normalized to CYPB expression and presented as fold change relative to pLXSN + ShCtl cells after one day (D1) of differentiation (n = 3). (**I**) Quantification of MyHC western blot from (G), normalized to CYPB expression and presented as fold change relative to pLXSN + ShCtl cells after two days (D2) of differentiation (n = 4). Bars are the mean ± SEM. Means with different letters or symbols are significantly different from one another, p < 0.05 (ANOVA, Tukey post-hoc test).

Given that *Id3* is the most abundantly expressed ID family member in myoblasts and was most upregulated by C/EBPβ (Figs 1A,D, 2B–D), we first examined a potential role for ID3 in the inhibition of myogenic differentiation by C/EBPβ. C/EBPβ-overexpressing myoblasts and empty virus controls were retrovirally transduced with shRNA targeting *Id3* mRNA (shId3) or with non-silencing shRNA (shCtl) and induced to differentiate. RT-qPCR confirmed both the overexpression of *Cebpb* and knockdown of *Id3* (Fig. 3A,B). While Id3 mRNA expression was reduced approximately 50% in pLXSN-controls by the *Id3*-targetting shRNA, the expression of *Id1* and *Id2* were not affected (Fig. 3B,C). Overexpression of C/EBPβ increased *Id3* expression in cultures transduced with the shCtl construct, and this was reduced approximately 80% by the shId3 construct (Fig. 3B). *Id1* expression was also significantly increased by C/EBPβ overexpression, but returned to baseline levels with addition of the shId3 construct (Fig. 3C). Given that the shId3 construct does not affect *Id1* and or *Id2* expression in the absence of C/EBPβ-overexpression, the reduction in *Id1* expression could be a consequence of enhanced differentiation, which is accompanied by downregulation of ID expression. To assess the efficacy of myogenic differentiation in these cultures, myosin heavy chain (MyHC) immunostaining was performed (Fig. 3D). Knockdown of *Id3* in empty vector controls increased the percentage of nuclei found in MyHC-expressing cells after 2 and three days in differentiation medium (Fig. 3D,E). Consistent with our previous findings^22^, the differentiation index (% nuclei in MyHC^+^ cells/total nuclei) was reduced when C/EBPβ was overexpressed and this inhibition was rescued when *Id3* was knocked down (Fig. 3D,E), restoring the differentiation index to the level of controls at both time points. However, loss of *Id3* failed to rescue fusion (the average number of myonuclei/MyHC + cells) in C/EBPβ-overexpressing cells (Fig. 3D,F) indicating that C/EBPβ has a role in negatively regulating cell fusion independently of ID3. Western blot analysis revealed that the knockdown of *Id3* expression in empty vector control cells (pLXSN) increased the expression of myogenin after one day in differentiating medium by ~2.5 fold (Fig. 3G,H). Additionally, while myogenin expression is decreased approximately 60% in C/EBPβ-overexpressing cells on day one of differentiation, this was rescued to the levels of pLXSN controls with loss of *Id3* (Fig. 3G,H). On day two of differentiation, myogenin expression was no longer statistically different with knockdown of *Id3*, resulting in a normalization of its expression across experimental conditions at this time point (Fig. 3H). However, the loss of *Id3* enhanced the expression of MyHC in pLXSN control cells and restored the defective MyHC expression observed in C/EBPβ-overexpressing cells on day two of differentiation (Fig. 3G,I).

Since ID1 protein levels are affected by overexpression of C/EBPβ, we assessed whether knockdown *Id1* could also rescue C/EBPβ-mediated inhibition of myogenic differentiation (Fig. S1). C2C12 myoblasts overexpressing C/EBPβ and their empty vector controls (pLXSN) were transduced to express a shRNA targeting *Id1* (shId1) or a non-targeting control (shCtl) (Fig. S1A). After 3 days of differentiation, myosin heavy chain immunostaining revealed that, in contrast to knockdown of *Id3*, knockdown of *Id1* did not enhance myogenic differentiation in pLXSN control cells as measured by the differentiation and fusion indices (Fig. S1B–D). However, the differentiation index of C/EBPβ-overexpressing cells was restored to normal levels with knockdown of *Id1*. Similar to shId3 knockdown, the knockdown of *Id1* failed to rescue the defective fusion index in C/EBPβ-overexpressing cells. These data highlight the dominant role of ID3 over the other ID family members, at least in normal cells, as only knockdown of *Id3* in control cells enhanced differentiation and fusion.

**Figure 4:**
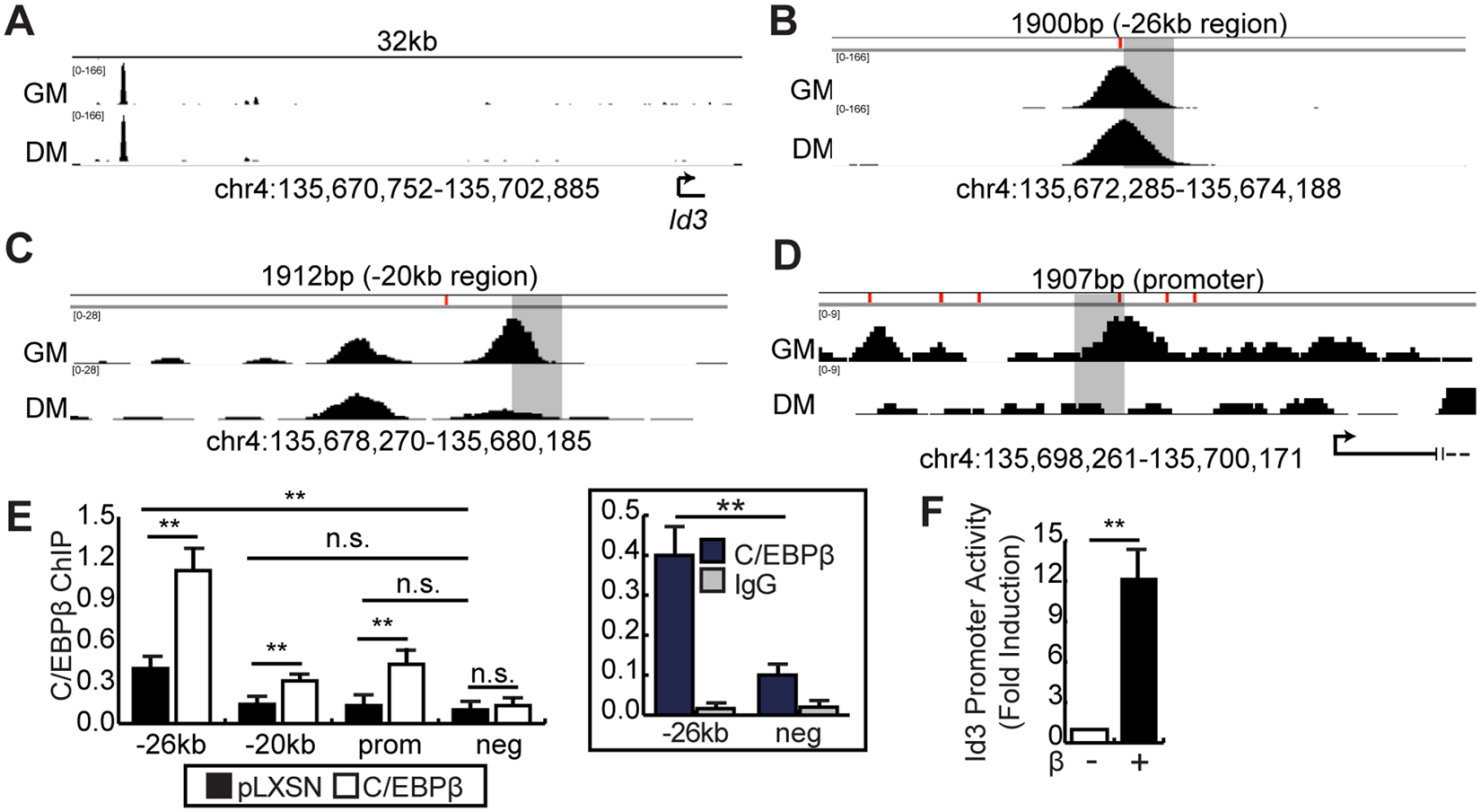
C/EBPβ is a direct transcriptional regulator of *Id3*. (**A**) *Id3* regulatory region (Chr4:135,670,752-135,702,885) with C/EBPβ (GSE36024) binding peaks indicated in proliferating (GM) and differentiating (DM) C2C12 myoblasts. C/EBPβ (GSE36024) binding peaks at (**B**) the −26kb upstream region of *Id3* (Chr4:135,672,285-135,674,188), (**C**) the −20kb upstream region of *Id3* (Chr4:135,678,270-135,680,185) and (**D**) the promoter region of *Id3* (Chr4:135,698,261-135,700,171). C/EBP motifs are indicated by vertical dash marks above each histogram and the targeted region by qPCR is highlighted in grey. (**E**) qPCR-ChIP analysis of C/EBPβ recruitment to the *Id3* regulatory regions in C2C12 cells retrovirally transduced with empty virus (pLXSN) or to express C/EBPβ and differentiated for three days. qPCR-ChIP data is shown as copy number as compared to a standard curve of 10% input of each condition. (n = 4). (**Boxed**) qPCR-ChIP data of C/EBPβ occupancy in pLXSN cells at the −26 kb region and the negative region presented with IgG control and shown as copy numbers in relation to the 10% input of each condition (n = 4). (**F**) *Id3* promoter activity in a transient transcription assay where the *Id3* promoter (−935 to + 13) drives expression of luciferase. Proliferating C2C12 cells were transiently transfected with the reporter construct and to express C/EBPβ and luciferase activity measured and shown relative to non-C/EBPβ expressing controls (n = 4). Bars are the mean ± SEM, **p < 0.01, n.s. is not significant (two-tailed Student’s t-test).

To determine if C/EBPβ directly regulates transcription of the *Id3* gene, we used published ChIP-seq signal tracks from proliferating and differentiating C2C12 myoblasts (GSE36024) to identify C/EBPβ occupancy peaks at the promoter and two additional upstream regions (Fig. 4A–D). *In silico* analysis of the regulatory region of *Id3* revealed that six putative C/EBP binding sites are located within −1 kb of the transcription start side (TSS), three of which are found within −300 bp of the TSS (Fig. 4D). We also found C/EBPβ motifs at approximately −26 kb and −20 kb from the TSS (Fig. 4B,C). ChIP-qPCR analysis in differentiating myoblasts retrovirally transduced with empty virus (pLXSN) or to overexpress C/EBPβ (β) confirmed the binding of C/EBPβ at the −26 kb upstream region (Fig. 4E and inset). C/EBPβ occupancy of the −20 kb and promoter region of *Id3* in empty virus control cultures was not significantly different from that of a negative region (Fig. 4E). However, in C/EBPβ-overexpressing cells, C/EBPβ recruitment at all *Id3* regulatory regions examined was enriched as compared to pLXSN (Fig. 4E) suggesting that overexpression, which occurs *in vivo* with sarcopenia and cachexia^28,29^, can force C/EBPβ onto elements that are not normally occupied. To confirm specificity, we repeated the ChIP in primary myoblasts isolated from a conditional null mouse where *Cebpb* is excised in muscle precursor cells (*Cebpb*^fl/fl^*Pax7*^CreER/wt^ (C/EBPβ^−/−^)) (Fig. S2A). In this experiments, C/EBPβ recruitment to the −26kb region of the *Id3* gene was significantly reduced in C/EBPβ^−/−^ cells when compared to WT controls, while other regions which did not show significant C/EBPβ recruitment were unaffected (Figs S2A, 4E). To confirm the direct regulation of *Id3* by C/EBPβ, we examined the ability of C/EBPβ to activate the *Id3* promoter in a luciferase reporter assay^30^. In cells transiently expressing C/EBPβ, *Id3* promoter activity was increased ~12 fold indicating that *Id3* is a novel direct transcriptional target of C/EBPβ in myoblasts (Fig. 4F).

Given that ID1 protein levels are increased with C/EBPβ overexpression, we examined C/EBPβ recruitment to the *Id1* promoter region. In contrast to the *Id3* promoter, where overexpression of C/EBPβ increased its recruitment, the *Id1* promoter was no different than a chromatin region devoid of C/EBPβ peaks (Fig. S2B).

### Loss of Id3 rescues myogenic differentiation in cancer cachexia

**Figure 5:**
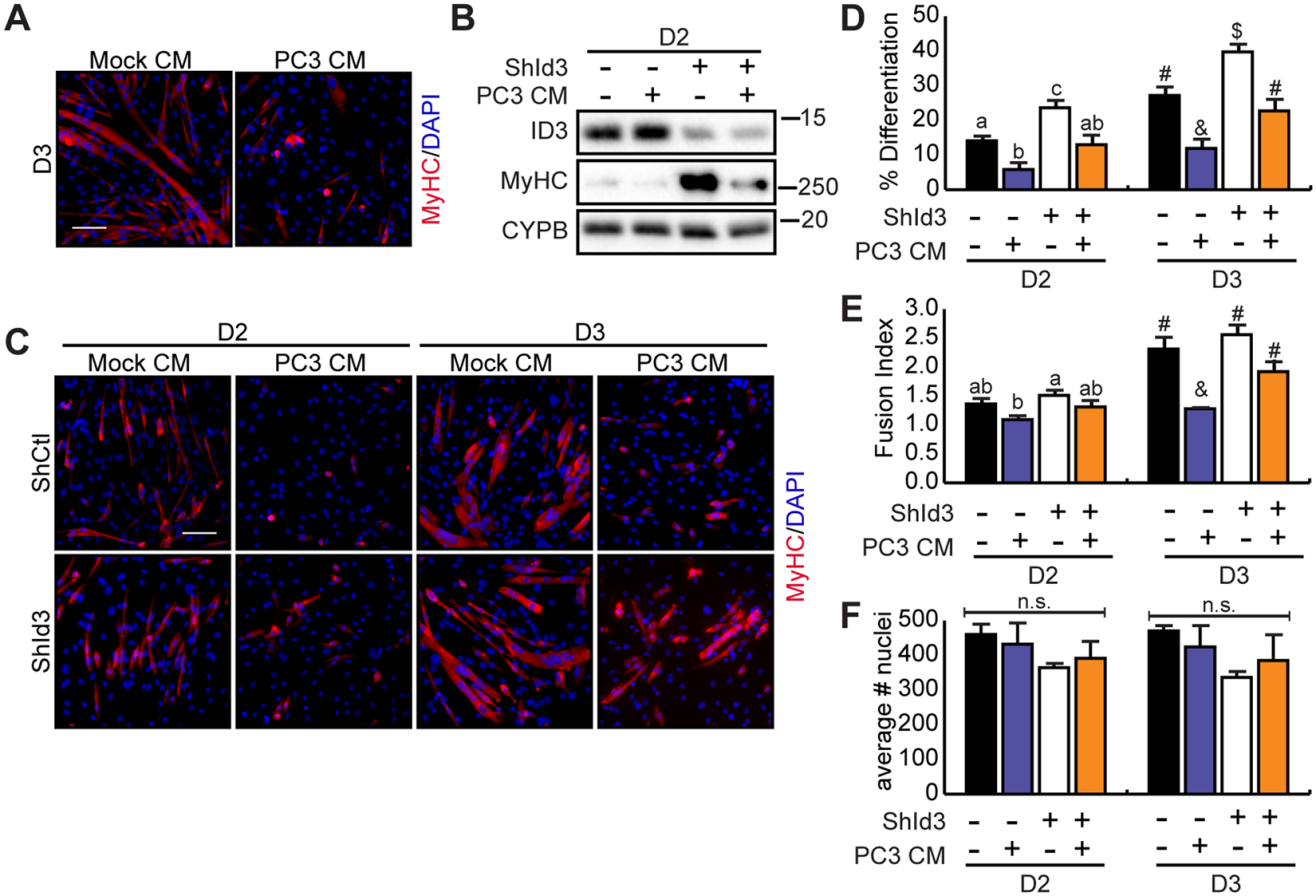
Loss of ID3 rescues myogenic differentiation in an *in vitro* model of cachexia. (**A**) Representative images of myosin heavy chain (red) immunostaining of C2C12 myoblasts that grown in C2C12-conditioned media (Mock CM) or PC3-conditioned media (PC3 CM) for 48 h then differentiated in fresh medium for three days. Nuclei are counterstained with DAPI (blue). Scale bar = 50 μm. (**B**) ID3 and myosin heavy chain (MyHC) protein expression in C2C12 cells retrovirally transduced to express a control shRNA or a shRNA directed against *Id3* (ShId3). Myoblasts were treated as in (A) and collected as differentiating cells (D2). (**C**) Myosin heavy chain immunostaining of C2C12 cells transduced as in (**B**) and treated and cultured as in (A). (**D**,**E**) Differentiation index (# of nuclei in MyHC-expressing cells/ total nuclei) and fusion index (average # of nuclei per MyHC + cell) for (C) (n = 4). (**F**) Average number of nuclei per field of view in cultures from (C). Data information: Bars are the mean ± SEM. Means with different letters or symbols are significantly different from one another, p < 0.05 (ANOVA, Bonferroni’s multiple comparison test).

We have previously shown that exposure of myoblasts to conditioned medium from cachexia-inducing tumours, can upregulate C/EBPβ expression and inhibit myogenic differentiation. Indeed, loss of C/EBPβ rescues myoblast differentiation in this model system^28^. Given that ID3 is downstream of C/EBPβ and loss of ID3 expression increases myogenin expression and promotes myogenic differentiation (Fig. 3), we asked if the knockdown of ID3 could rescue differentiation in an *in vitro* cancer cachexia model^31^. C2C12 myoblasts were grown in conditioned media (CM) from a cachexia-inducing prostate cancer cell line (PC3) or proliferating C2C12 cells (mock) for 48 hours prior to induction to differentiate. Differentiation was then induced in the absence of conditioned medium for up to 3 days. Inhibition of myogenic differentiation in cells pre-treated with PC3-conditioned media was confirmed by MyHC staining (Fig. 5A). We next asked if knockdown of ID3 could rescue the defective differentiation seen in myoblasts pre-treated with PC3-conditioned media. Under differentiation conditions, knockdown of ID3 stimulated myosin heavy chain protein expression in control cells and in cells pre-cultured in PC3-conditioned media as compared to control shRNA (Fig. 5B). Indeed, pre-treatment with PC3-conditioned medium inhibited differentiation in shCtl cells but knockdown of ID3 rescued this defect in differentiation, most prominently on day 3 (Fig. 5C,D). Furthermore, while pre-treatment with PC3 conditioned medium reduced myotube size in shCtl cells on day 3, this effect was rescued in cultures lacking ID3, suggesting that the blockade in myogenic differentiation observed in cancer cachexia is mediated through misexpression of ID3 (Fig. 5C,E). Pre-treatment with PC3-conditionned medium did not influence cell density in these experiments, nor did knockdown of *Id3* (Fig. 5F).

## Discussion

Herein, we demonstrate that C/EBPβ can upregulate *Id1* and *Id3* but not *Id2* expression in myoblasts. We find that *Id3* is the most abundant Id family member expressed in myoblasts and the stimulation of *Id3* expression by ectopic C/EBPβ is more pronounced than that of *Id1*. We demonstrate that C/EBPβ binds the regulatory region of *Id3* (promoter and two upstream regions) and is a direct target of C/EBPβ in myoblasts. Moreover, the knockdown of *Id3* rescues the inhibition of myogenic differentiation in C/EBPβ-overexpressing cells, placing *Id3* as a major effector of C/EBPβ action in this system.

While C/EBPβ is a known transcriptional regulator of both *Id1* and *Id2* in the immune system^24–26^, changes in C/EBPβ expression did not affect *Id2* expression in myogenic cells, suggesting cell-type specific regulation of these genes by C/EBPβ. Interestingly, *Pax7* is also a direct target of C/EBPβ^22^ and PAX7 has been shown to also regulate *Id3* expression^30^, suggesting that C/EBPβ can regulate *Id3* expression directly as well as indirectly through PAX7.

While all ID proteins bind E-proteins with high affinity and thus can interfere with the actions of bHLH transcription factors, in muscle, their effects are not entirely overlapping. For example, ID1 and ID2 appear to inhibit MYOD and MYF5 action but not that of MYOG or MRF4, while ID3 showed a weak inhibition of all MRFs^32^ suggesting that ID3 can function both in early and late myogenic differentiation if it is expressed, as occurs in cells overexpressing C/EBPβ and in our model of cancer cachexia. While the overexpression of ID2 but not ID3 in myogenic cells has been shown to inhibit the induction of myogenin expression^12,14^ we find that knockdown of *Id3* increases myogenin protein expression even in the presence of ectopic C/EBPβ. Further, we have shown that C/EBPβ can reduce MYOD activity, an upstream regulator of myogenin^22^ though whether this effect is mediated directly by ID3 remains to be investigated. As such, our findings suggest that the misexpression of ID3 could therefore have a more potent impact of the progression of differentiation by targeting the late regulators of differentiation.

The shRNA used to knockdown ID3 expression is specific and failed to decrease the mRNA expression of *Id1* or *Id2* in control cultures. However, in the presence of ectopic C/EBPβ, the expression of the *Id3*-targeting shRNA did reduce *Id1* mRNA, suggesting that the loss of ID3 in these cells restores the myogenic differentiation program including the downregulation of ID1 expression. Indeed, knockdown of *Id1* in C2C12 cells overexpressing C/EBPβ was able to rescue differentiation but not fusion, but was without effect in control cells. While these findings would suggest that ID3 is the dominant ID protein in myoblasts under normal physiological conditions, in disease states where C/EBPβ expression is induced in myoblasts, for example cachexia, both *Id1* and *Id3* upregulation are likely to contribute. While we did not see recruitment of C/EBPβ to the *Id1* promoter, this analysis did not include possible enhancer regions. Another condition that is associated with poor muscle regeneration and differentiation is sarcopenia, the loss of muscle mass that accompanies aging. It has been reported that the expression of *Cebpb* in primary myoblasts increases with mouse age (3-week, 3-month and 18-month of age)^33^, and *Cebpb* is considered part of a molecular signature of muscle aging^29^. Interestingly, analysis of the raw microarray data set from Price, Von Maltzahn *et al*.^33^, revealed that along with upregulation of *Cebpb* in aging primary myoblasts, there is upregulation of *Id1* and *Id3*, but not *Id2*, consistent with our findings. These findings suggest that ID3 is an interesting therapeutic target to restore differentiation function in aged myoblasts.

Despite restoring myogenic marker expression, knockdown of *Id3* was not able to restore fusion, suggesting that C/EBPβ acts to limit myotube size in a mechanism that is independent of ID3. In cancer cachexia however, where treatment of proliferating myoblasts with conditioned medium from the PC3 tumour is known to transiently upregulate C/EBPβ expression and to inhibit myogenic differentiation^28^, knockdown of ID3 rescues both differentiation and fusion. Indeed, given the design of the experiment with pre-treatment causing differentiation defects, it is possible that at the time of fusion, the cells have recovered from the cachectic insult. Examination of this in co-culture experiments would clarify the role for ID3 in this context.

## Experimental Procedures

### Cell Culture

Mouse primary myoblasts were isolated and cultured as previously described^22^. Briefly, hindlimb muscles of adult mice (aged 6–8 weeks) were dissected and digested with collagenase/Dispase (Roche). Isolated cells were then pre-plated to remove fibroblasts and primary myoblasts were maintained on matrigel-coated plates in Dulbecco modified eagle medium DMEM (Wisent) supplemented with 20% fetal bovine serum (FBS) (Wisent), 10% horse serum (HS) (Sigma), 10 ng/ml basic fibroblast growth factor (bFGF) and 2 ng/ml hepatocyte growth factor (HGF) (Peprotech). To induce differentiation, confluent cultures were switched to differentiation media (DMEM, 2% FBS and 10% HS). *Primary* myoblasts from *Cebpb*^fl/fl^*Pax7*^wt/wt^ (WT) or *Cebpb*^fl/fl^*Pax7*^*CreER*/wt^ (cKO) mice isolated as previously described^22^, were treated with 2 µM 4-OH tamoxifen (Sigma) for 48 h to induce excision of *Cebpb* in Cre-expressing cells. C2C12 myoblasts (ATCC) were grown in DMEM supplemented with 10% FBS (GM, growth media) and differentiation was induced by switching confluent cells to DMEM supplemented with 2% HS. PC-3 human prostate cancer cells (ATCC) were maintained in Roswell Park memorial institute medium (RPMI 1640) (Sigma) supplemented with 10% FBS. For *in vitro* cachexia experiments, conditioned media was collected from 90% confluent PC-3 cells after two days and added to C2C12 growth media (1:1 ratio) with fresh medium^31^. After two days in conditioned media, C2C12s were switched to fresh differentiation media.

All animal experiments were approved by the University of Ottawa Animal Care Committee and all procedures were performed in accordance with the regulations set out by the Canadian Council on Animal Care.

### Retroviral Infection

Replication incompetent retroviruses were generated in Phoenix Ampho packaging cells (ATCC) as previously described^22^ using pLXSN-C/EBPβ plasmid. Following retroviral infection of C2C12 myoblasts with empty virus (pLXSN) or virus to express C/EBPβ (pLXSN-C/EBPβ), pooled stable cells were selected based on neomycin (Wisent) resistance. Knockdown of *Id3* was accomplished by retrovirally transducing C2C12 myoblasts with a sequence targeting *Id3* (pRS-shId3, Origene, TR501033), Id1 (pRS-shId1, Origene, TR511687) or a non-targeting control sequence (shCtl, Origene) and stable cells generated based on puromycin (Wisent) resistance.

### Immunofluorescence

Cells were fixed in ice-cold 100% methanol and then permeabilized with PBS containing 0.5% Triton X-100 (Bioshop). Cells were incubated in primary antibody (MF20, DSHB) overnight at 4 °C followed by secondary antibody (anti-mouse Cy3, Jackson ImmunoResearch) incubation. Nuclei were counterstained with 0.5 μg/ml DAPI. Images were captured using a Zeiss AxioObserver D1 microscope (Zeiss) at the 10X objective. ImageJ software was used for the quantitative analysis of MyHC stained cells. Representative images were cropped using Adobe Photoshop.

### Western Analysis

Whole cell extracts were prepared from primary myoblasts or C2C12 myoblasts. Equal amount of proteins were resolved on a 15% SDS-PAGE gel and then transferred to a PVDF membrane (Bio-Rad). Membranes were then probed with the following primary antibodies: C/EBPβ (E299, ab32358), ID1 (Biocheck, BCH-1/37-2), ID3 (Biocheck, BCH-4/17-3), myogenin (F5D, DSHB), MyHC (MF20, DSHB) and Cyclophilin B (ab16045). The ChemiDoc^TM^ MP system (Bio-Rad) was used to detect chemiluminescence. Resultant images were cropped in Adobe Photoshop.

### Real-time quantitative PCR

RNA was isolated using RNeasy kit (Qiagen) as per manufacturer’s protocol. The remaining DNA was digested with DNase (Ambion) and DNA-digested RNA was used to make cDNA using iScript™ cDNA Synthesis Kit (Bio-Rad) following the manufacturer’s protocol. RT-qPCR reactions were performed using iTaq^TM^ Universal SYBR^®^ Green Supermix (Bio-Rad) on the CFX96 platform (Bio-Rad). Relative transcript expression was calculated using the ΔΔCt method after normalizing each to 18 S rRNA. The primer sequences used in this study are as followed: *Cebpb-F*: TCGAACCCGCGGACTGCAAG *Cebpb-R*: CGACGACGACGTGGACAGGC *Id1-F*: GAGGCGGCATGTGTTCCA *Id1-R*: CTCTGGAGGCTGAAAGGTGG *Id2-F*: GGACTCGCATCCCACTATCG *Id2-R*: GATGCCTGCAAGGACAGGAT *Id3-F*: AGCTCACTCCGGAACTTGTG *Id3-R*: GTTCAGTCCTTCTCTCGGGC *Myog-F*: ATCGCGCTCCTCCTGGTTGA *Myog-R*: CTGGGGACCCCTGAGCATTG *18s-F*: CGCCGCTAGAGGTGAAATC *18s-R*: CCAGTCGGCATCGTTTATGG

### Chromatin Immunoprecipitation

C2C12 myoblasts were crosslinked with 1% formaldehyde for 30 min then sonicated for 30 cycles (30 sec ON/OFF) using Diagenode Bioruptor^®^. Equal amounts of chromatin was used to perform Immunoprecipitation (ChIP) analysis as previously described^23^ using C/EBPβ (C-19, Santa Cruz Biotech, Sc-150) or rabbit polyclonal IgG (Invitrogen). Data are presented as copy numbers as compared to a standard curve that was generated using 10% input of each sample. The primer sequences used are as followed: *Id3*_−26kb (chr4: 135, 672, 285-135, 674, 188) F:GGCTGTTCGTTGACCTTGTTT R: AGGGAATCGTGACGGTTGG, *Id3*_−20 kb (chr4: 135, 678, 270-135, 680, 185) F: TTCGAAAGGCTTCCGGGCTAA R: TCCCTGCGACCCAAAGCTTAC, *Id3*_promoter (chr4: 135, 698, 261-135, 700, 171) F: AGTTCTCGGTGGAAACGGTC R:CTAGGCGCTGAGATTGCAGA, *Id1*_promoter (chr2: 152, 736, 188-152, 736, 308) F: TTTGAACGTTCTGAACCCGC R: GGCTGAGAACAGAGTGTGGG, Negative region (chr11: 71, 360, 398-71, 360, 930) F: TCCCAGCTCACAGGCTAGAA R: AATGCAGAGCAGAAGGGGTC.

### Luciferase Assay

The −934/ + 13 bp *Id3*-luciferase reporter construct was kindly provided by Dr. Andrew Lasser^30^. Briefly, C2C12 cells were transiently transfected with the Id3-luc reporter construct and a constitutively active RSV-β-galactosidase reporter in the presence or absence of mammalian expression plasmid for C/EBPβ using FuGENE HD (Promega). Cells were collected 48 h post-transfection to assess luciferase activity using the Dual-Luciferase Reporter Assay kit (Promega) according to manufacturer’s instructions. Luciferase activity was measured using a Monolight 2010 luminometer (Analytical Luminescence laboratory) and corrected for transfection efficiency with β-gal activity.

### Statistical Analysis

Two means were compared by Student’s t-test for a minimum of three biological repeats. For multiple means, one-way or two-way ANOVA was conducted with appropriate post hoc test. *p < 0.05, **p < 0.01, ***p < 0.001. Other comparisons are explained in the relevant legends. For multiple comparisons, means indicated by different letters or symbols are statistically different from one another with a p < 0.05 as a minimum cutoff.

## Electronic supplementary material

Supplemental Information

## Data Availability

All data generated or analyzed during this study are included in this published article (and its Supplementary Information files)
