# Supplementary material for: CCAAT/Enhancer Binding Protein β inhibits myogenic differentiation via ID3
Source: Sci Rep. 2018 Nov 9;8:16613. doi: 10.1038/s41598-018-34871-0 (PMC6226455; doi:10.1038/s41598-018-34871-0)

Supplementary Information

CCAAT/Enhancer Binding Protein β inhibits myogenic differentiation via ID3

Hamood AlSudais, Neena Lala-Tabbert and Nadine Wiper-Bergeron

CONTENTS:

(1) Supplementary legends for Figures S1 and S2

(2) Supplementary Figure S1

(3) Supplementary Figure S2

(4) Uncropped Western blots

Supplemental Legends

Figure S1. Knockdown of Id1 rescues differentiation but not fusion in C/EBPβ-overexpressing cells. (A) Representative western blot of C/EBPβ and ID1 expression in C2C12 myoblasts transduced to express C/EBPβ (β) or with empty virus (pLX) and a shRNA targeting *Id1* (shId1) or a non-targeting control (shCtl). Cyclophilin B (CYPB) is a loading control. **(B)** Immunostaining for myosin heavy chain (MyHC) in cultures derived as in (A) and differentiated for 3 days. DAPI stains nuclei blue. Scale bar = 50μm. **(C)** Differentiation index (#nuclei in MyHC+ cells/ total nuclei) in cells differentiated as in (B). **(D)** Fusion index (average # nuclei/MyHC+ cells). Bars are means + SEM, n=4. Means indicated with different letters are significantly different from one another, with a minimum cut-off of $p<0.05$.

Figure S2. (A) qPCR-ChIP analysis of C/EBPβ recruitment to the *Id3* regulatory regions in C/EBP$\beta^{-/-}$ (cKO) and WT control primary myoblasts and differentiated for one days. Recruitment is shown relative to IgG non-specific pulldown control. (n=2). Western blot validating C/EBPβ expression is shown as an inset. Cyclophilin B (CYPB) is a loading control. **(B)** qPCR-ChIP analysis of C/EBPβ recruitment to the *Id3* promoter, the *Id1* promoter and a negative region devoid of C/EBP response elements in C2C12 cells retrovirally transduced with empty virus (pLXSN) or to express C/EBPβ and differentiated for three days. qPCR-ChIP data is shown as copy number as compared to a standard curve of 10% input of each condition. **$p<0.01$, n=4. n.s. is not significant.

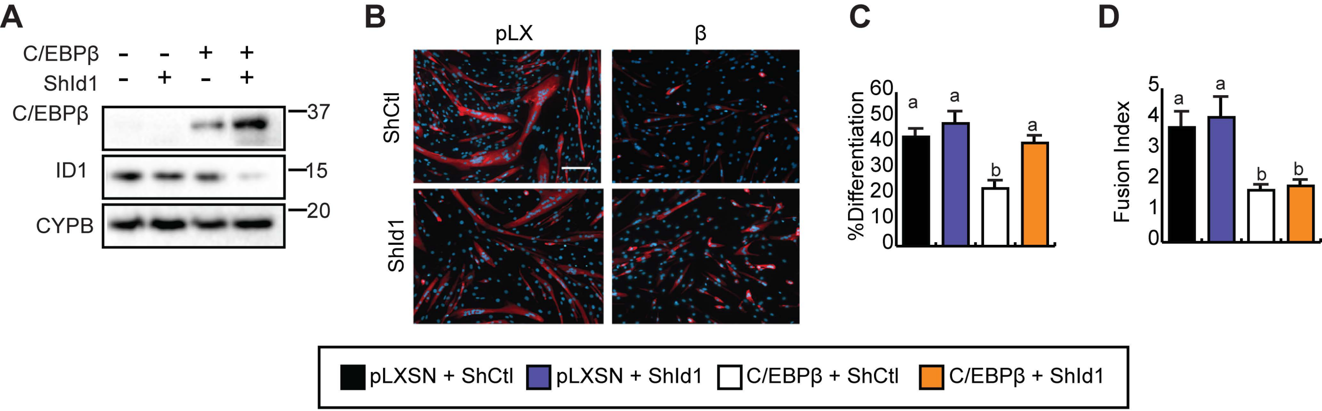

FIGURE S1. AlSudais et al.

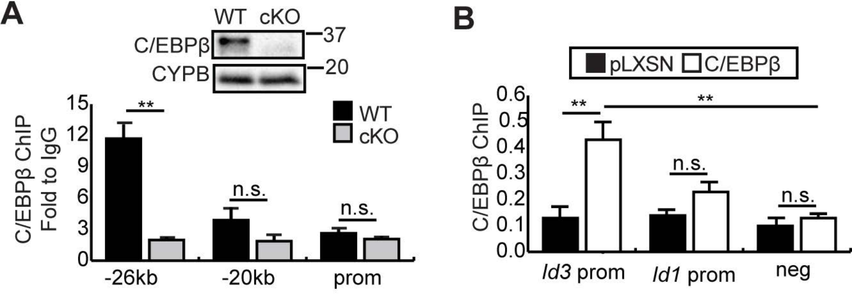

FIGURE S2. AlSudais et al.

Uncropped blots, Figure 1C

C/EBPβ

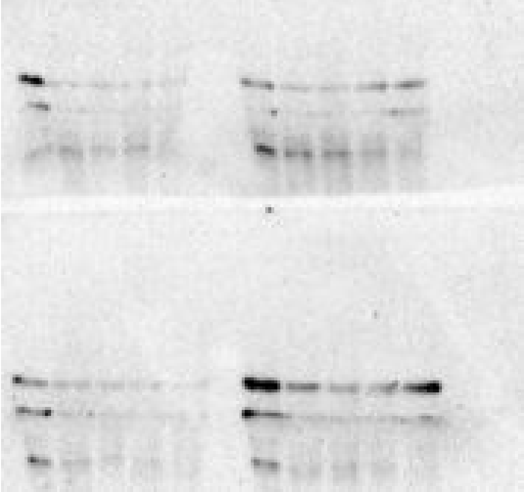

CYPB

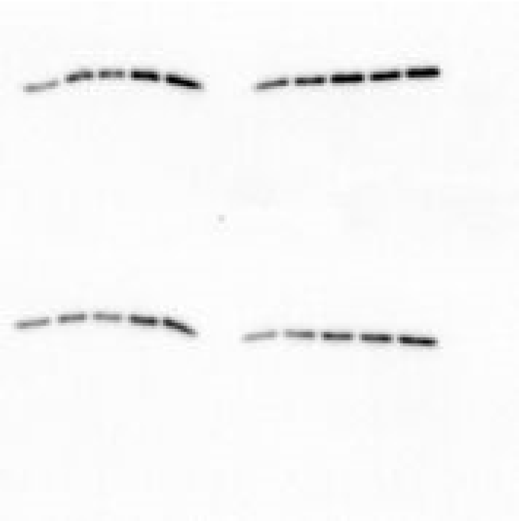

myogenin

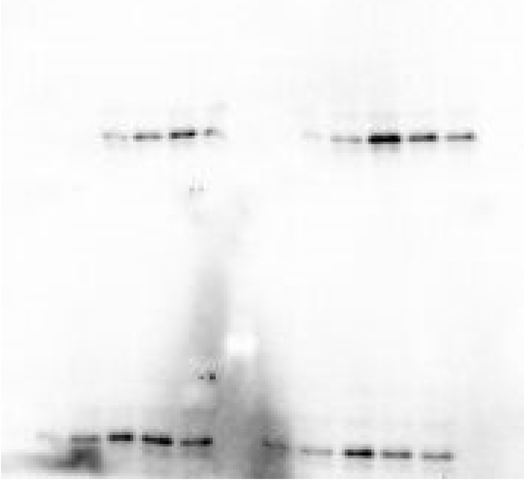

ID1

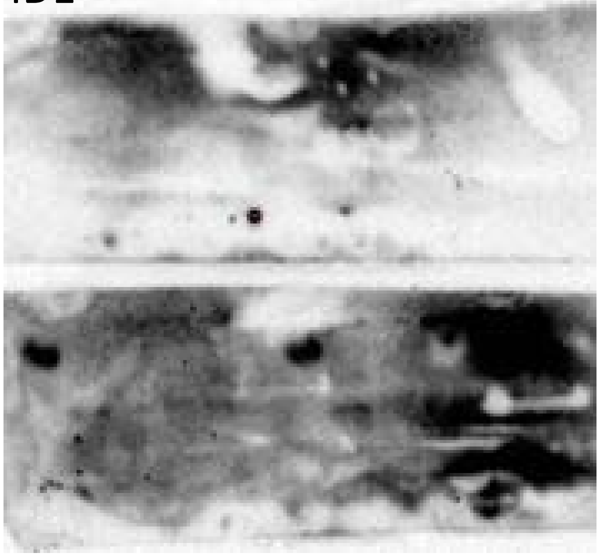

ID3

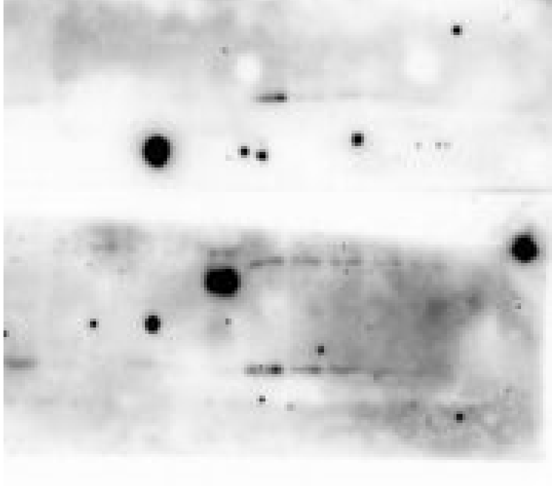

MyHC

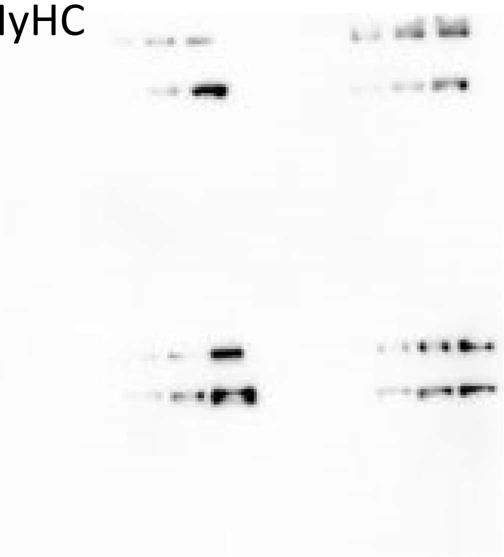

Uncropped blots, Figure 1F

C/EBPβ

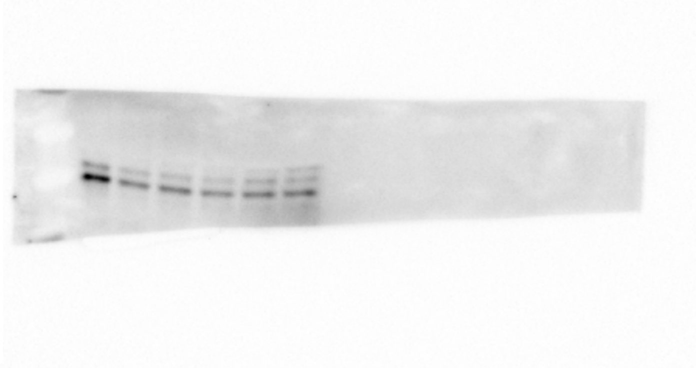

CyPB

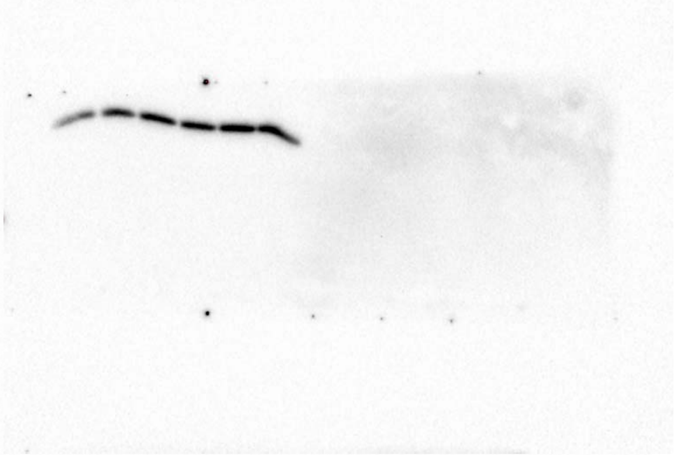

ID1

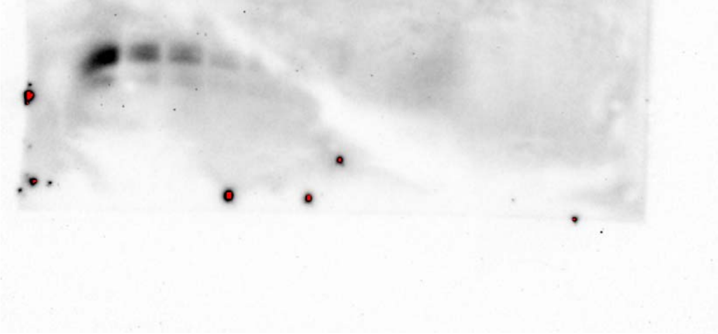

ID3

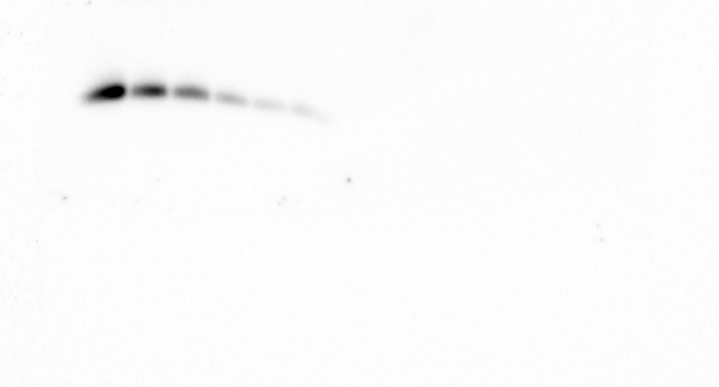

MyHC

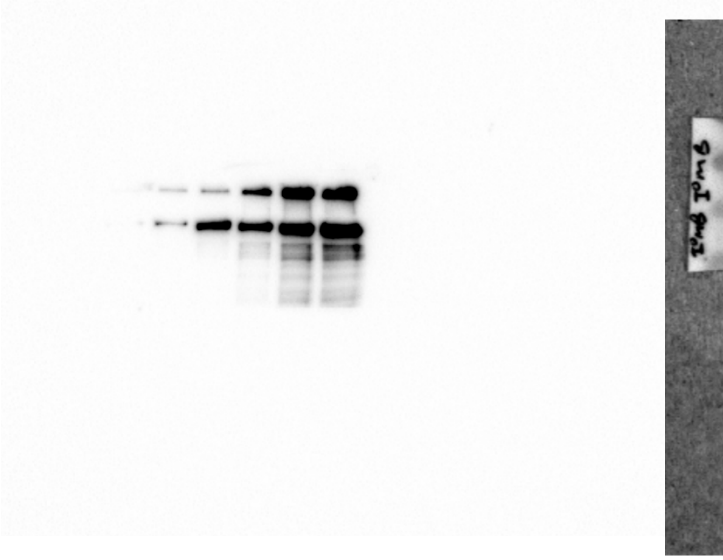

Myogenin

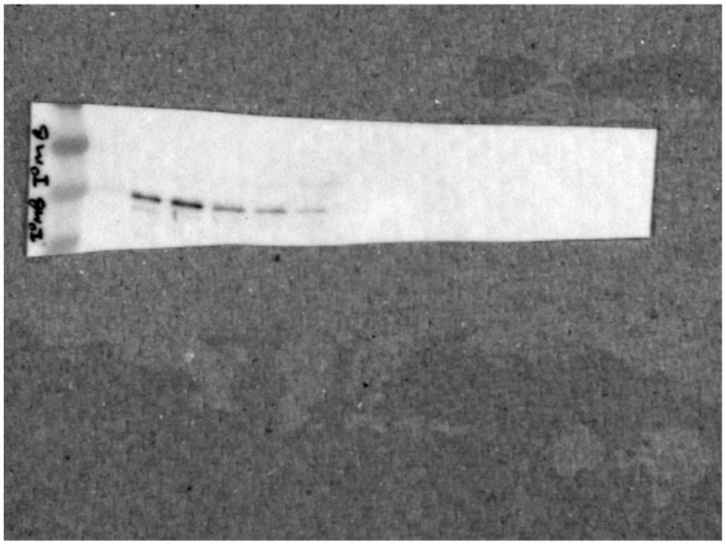

Uncropped Westerns, Figure 2A

CyPB

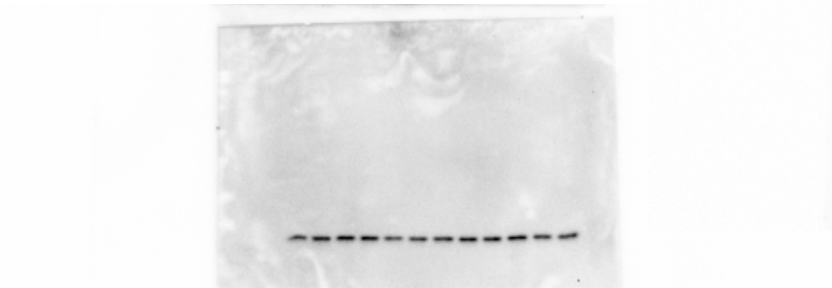

C/EBPβ

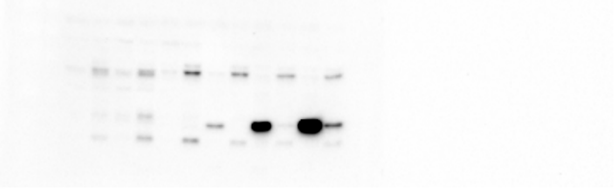

Uncropped Westerns, Figure 2C

ID1 ID3

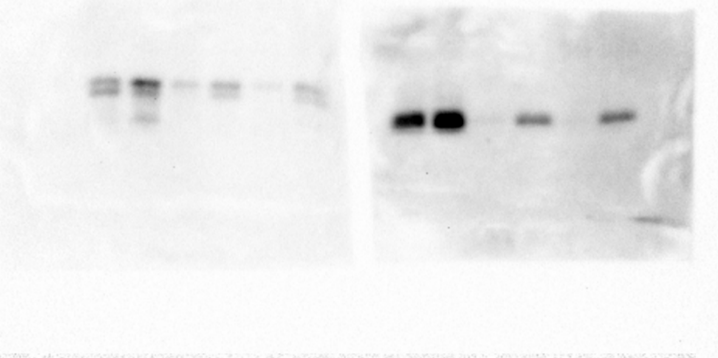

C/EBPβ

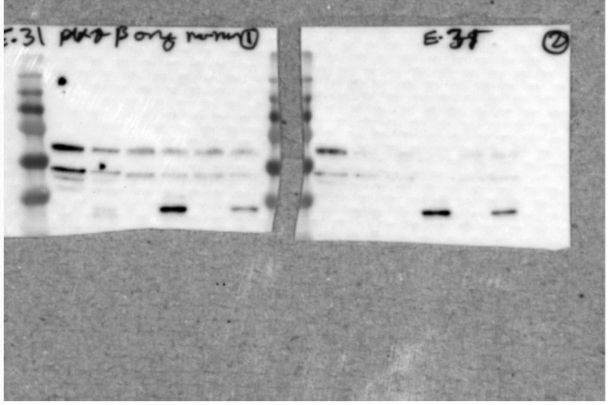

MYOG

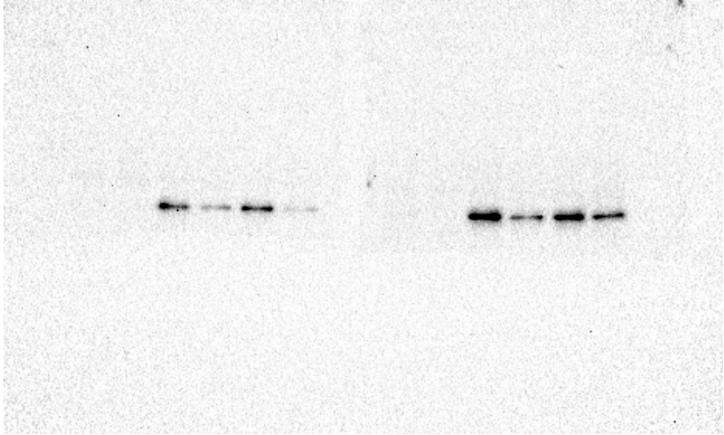

CyPB

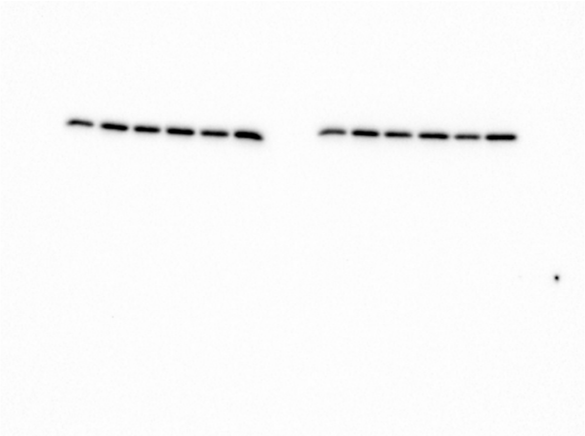

Uncropped Westerns, Figure 3G

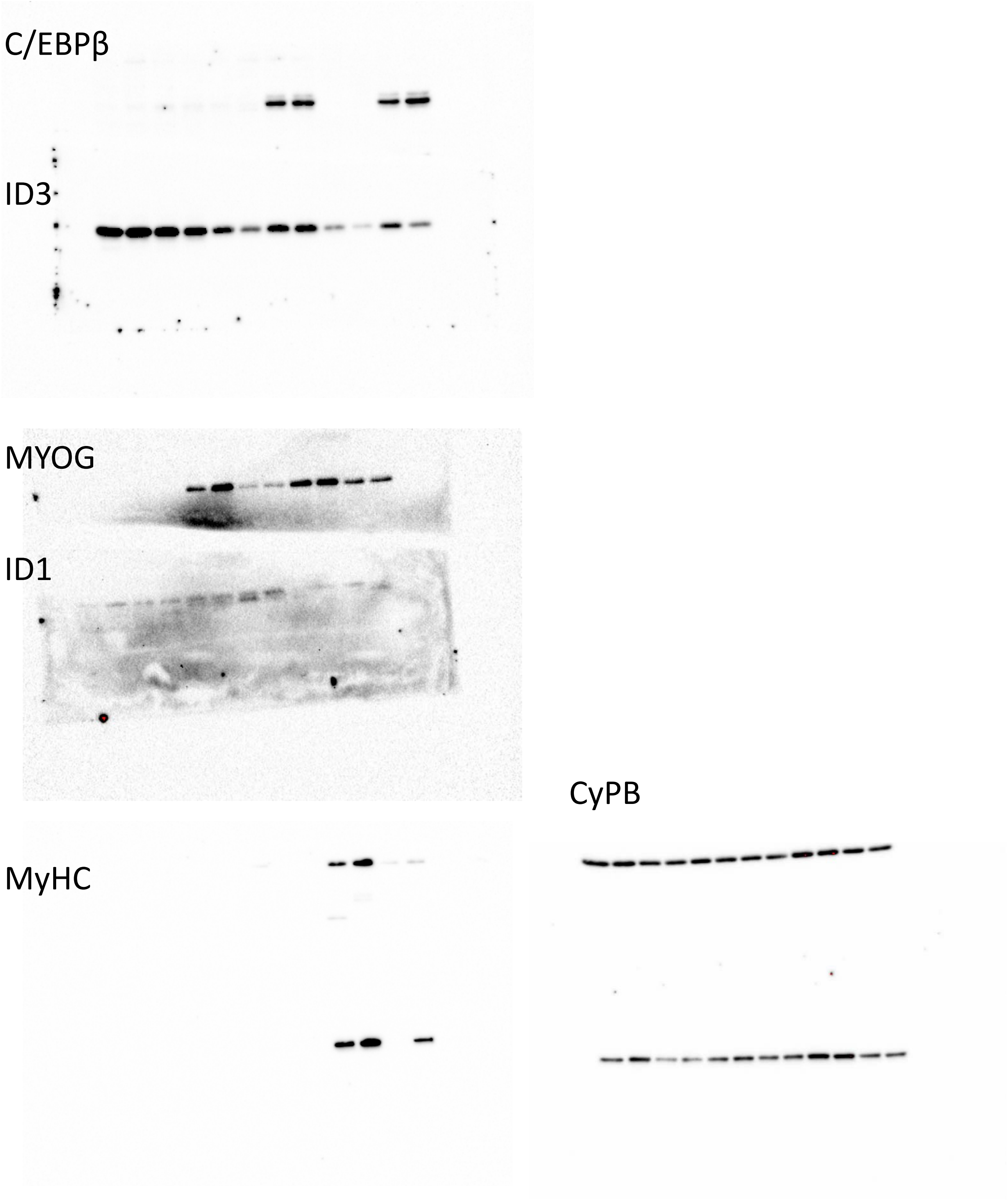

Uncropped Westerns, Figure 5B

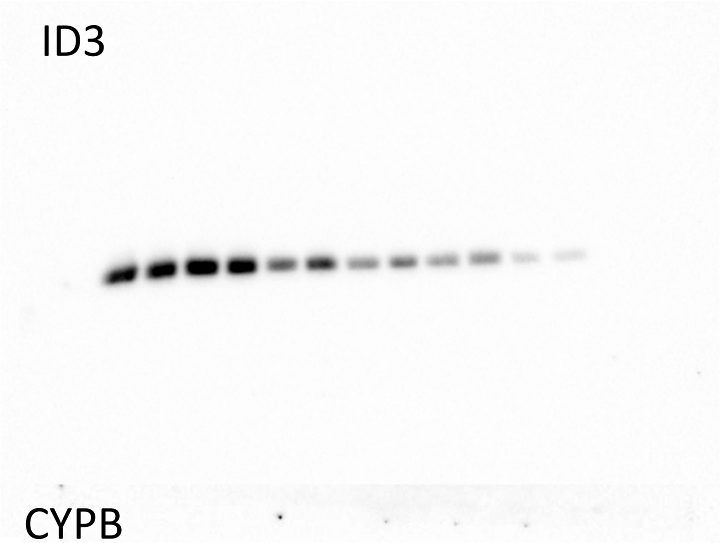

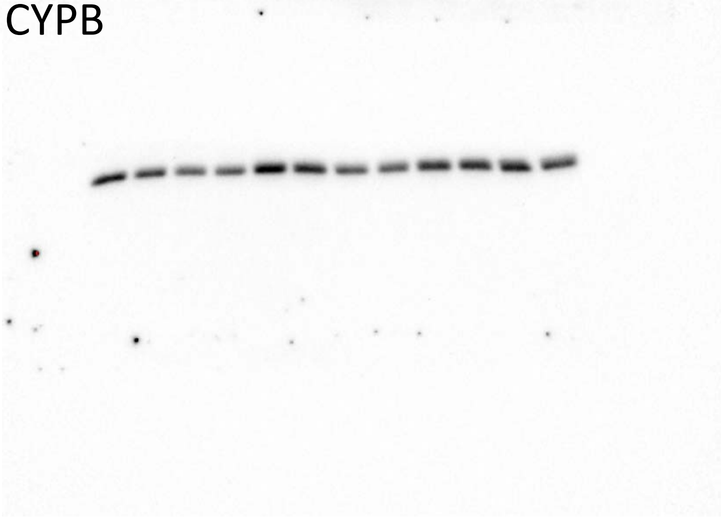

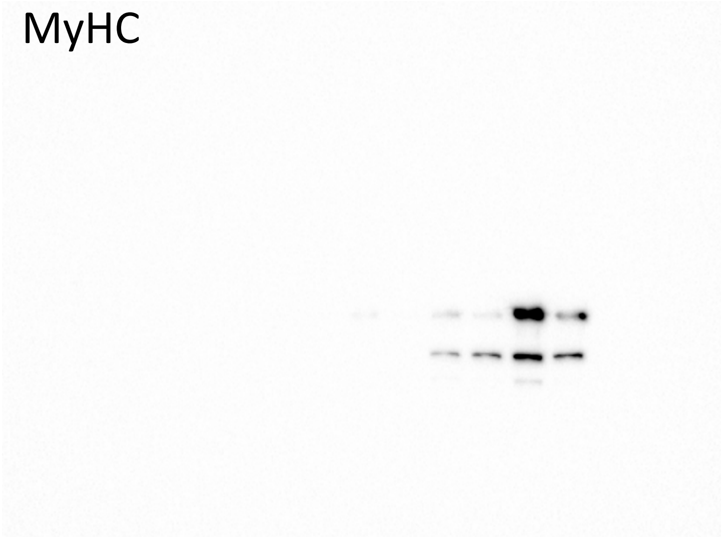

Uncropped Western blots Figure S1A

C/EBPβ

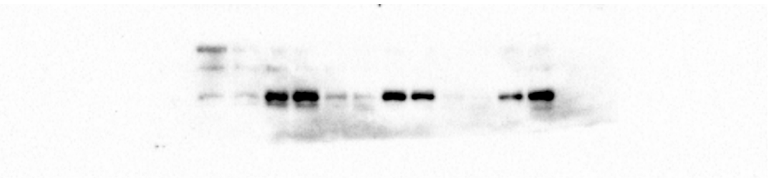

CYPB

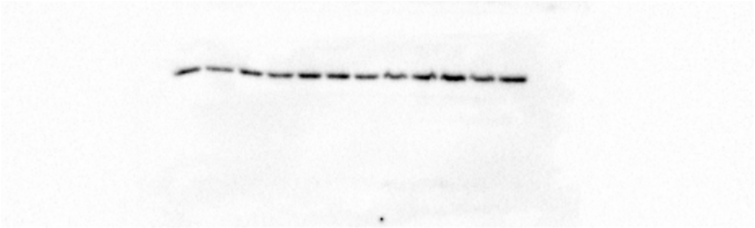

ID1

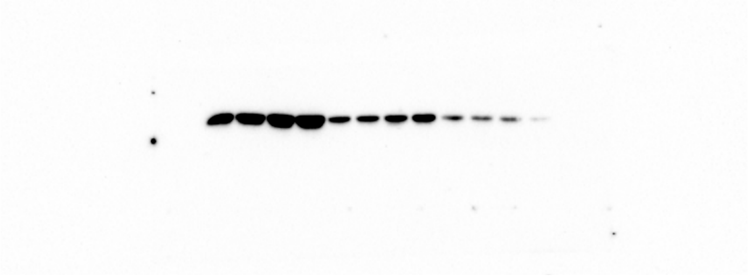

Uncropped Westerns, Figure S2A

C/EBPβ

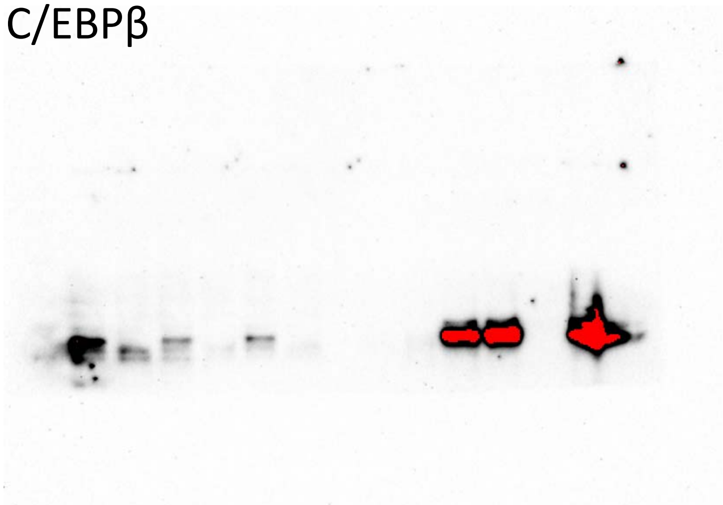

CYPB

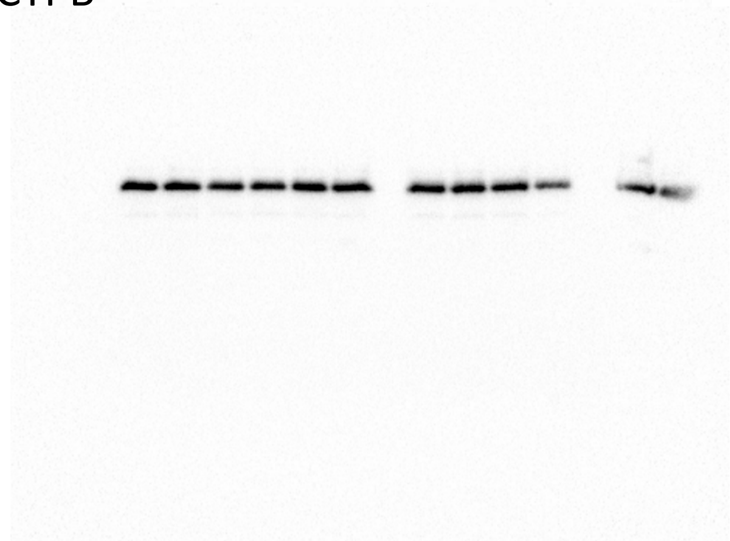

Supplement: Supplementary file 1 — Supplemental Information [file 41598_2018_34871_MOESM1_ESM.pdf]
